# Family history and breast cancer risk for Asian women: a systematic review and meta-analysis

**DOI:** 10.1186/s12916-023-02950-3

**Published:** 2023-07-03

**Authors:** Heran Wang, Robert J. MacInnis, Shuai Li

**Affiliations:** 1 Centre for Epidemiology and Biostatistics, Melbourne School of Population and Global Health, The University of Melbourne, 207 Bouverie Street, Carlton, VIC 3053 Australia; 2 China Astronaut Research and Training Centre, Beijing, 100094 China; 3 Cancer Epidemiology Division, Cancer Council Victoria, Melbourne, VIC 3004 Australia; 4 Centre for Cancer Genetic Epidemiology, Department of Public Health and Primary Care, University of Cambridge, Cambridge, CB1 8RN UK; 5 Precision Medicine, School of Clinical Sciences at Monash Health, Monash University, Clayton, VIC 3168 Australia; 6 Murdoch Children’s Research Institute, Royal Children’s Hospital, Parkville, VIC 3051 Australia

**Keywords:** Breast cancer, Family history, Asia, Ethnicity, Familial risk, Genetic susceptibility, Systematic review, Meta-analysis

## Abstract

**Background:**

Studies of women of European ancestry have shown that the average familial relative risk for first-degree relatives of women with breast cancer is approximately twofold, but little is known for Asian women. We aimed to provide evidence for the association between family history and breast cancer risk for Asian women by systematically reviewing published literature.

**Methods:**

Studies reporting the familial relative risk of breast cancer for Asian women were searched in three online databases and complemented by a manual search. Odds ratios (ORs) for the association between family history and breast cancer risk were pooled across all included studies and by subgroups in terms of the type of family history, age, menopausal status and geographical region.

**Results:**

The pooled OR for women who have a first-degree relative with breast cancer was 2.46 (95% confidence interval [CI]: 2.03, 2.97). There was no evidence that the familial risk differed by the type of affected relative (mother versus sisters), the woman’s age (< 50 years versus ≥ 50 years), menopausal status (pre versus post) and geographical region (East and Southeast Asia versus other regions) (all *P* > 0.3). The pooled ORs for women of Asian ancestry with a family history in any relative were similar for those living in non-Asian countries (2.26, 95% CI: 1.42, 3.59) compared with those living in Asian countries (2.18, 95% CI: 1.85, 2.58).

**Conclusions:**

Family history of breast cancer is associated with an approximately twofold relative risk of breast cancer for Asian women, which is of similar magnitude to that observed for women of European ancestry. This implies that similar familial factors are implicated in breast cancer risk between women of European and Asian ancestries. Genetic factors are likely to play a substantial role in explaining the breast cancer familial risk for Asian women, as similar risks were observed across different living environments and cultures.

**Supplementary Information:**

The online version contains supplementary material available at 10.1186/s12916-023-02950-3.

## Background

Family history is a strong risk factor for breast cancer. Previous studies have shown that the relative risk of breast cancer associated with an affected first-degree relative is approximately twofold, and the risk is higher when the number of affected relatives is greater, the relatives’ age at diagnosis is younger and the women’s age is younger [1–3]. Moreover, family history, as one of the essential predictors, has been included in the widely used breast cancer risk models, such as the Breast and Ovarian Analysis of Disease Incidence and Carrier Estimation Algorithm model (BOADICEA) [4], BRCAPRO [5], Breast Cancer Risk Assessment Tool (BCRAT) [6] and International Breast Cancer Intervention Study model (IBIS) [7].

It is worth noting that the risk association mentioned above and the development of these risk models are mainly based on data from women of European ancestry. Breast cancer incidence and burden for Asian women have been increasing [8], and understanding the familial risk of breast cancer for Asian women could help reduce the burden by providing evidence for the causes of familial risk (both genetic and environmental) [9–11] and for risk prediction based on familial history [4–7]. The risk association for breast cancer family history found in women of European ancestry may not be applicable to Asian women because of the substantial differences in genetic background, socio-economic profile, lifestyle and culture between Asian and European ancestry women [12].

Some reviews investigated breast cancer risk factors for women living in Asia but only included a few Asian countries (Malaysia, Indonesia, China, India and Korea) regarding family history [13, 14]; therefore, their findings might not be applicable to the whole Asian population. Furthermore, there is a lack of knowledge about breast cancer familial risk for women of Asian ancestry living in non-Asian countries. We aimed to investigate the association between family history and breast cancer risk for Asian women using a systemic review approach. To provide comprehensive evidence which can be generalised widely, women of Asian ancestry in this review are defined as those who live in Asian countries in the United Nations geoscheme as well as those who have Asian ethnicity (i.e. people having origins in the original peoples of the Asian countries) and live in non-Asian countries.

## Methods

This study design is a systematic review with a meta-analysis, which followed the Preferred Reporting Items for Systematic Reviews and Meta-Analysis Statement (PRISMA) [15]. This systematic review was registered at the protocol stage on an international prospective register of systematic reviews (PROSPERO; CRD42021262986) [16].

### Data sources

We searched for studies in three online databases including PubMed, Embase and Web of Science using Medical Subject Headings (MeSH) and Embase Subject Headings (Emtree) from the earliest publication date. Three search strategies were used: (1) (breast neoplasm) AND (family history) AND (Asia), (2) (breast neoplasm) AND (risk factor) AND (Asia) and (3) (breast neoplasms) AND (risk factors) AND (race OR ethnicity) (see Additional file 1: Literature search methods for more details). The first strategy aimed to find studies for Asian women that primarily investigated family history as a breast cancer risk factor, and the second strategy aimed to find studies for Asian women that investigated multiple breast cancer risk factors but did not report family history as the primary result, whilst the third strategy aimed to find studies about ethnicity-specific breast cancer risk factors that potentially included Asian women. Note that it is possible that a study can be found by all the strategies. We also complemented the search by screening the reference lists of the included studies. Titles and abstracts were reviewed first, where clearly irrelevant papers were excluded. After that, we reviewed the full text of the papers of the potentially relevant abstracts. Two investigators (H.W. and S.L.) independently conducted the literature search and study selection, and any disagreement was resolved by discussion and consensus.

### Study selection criteria

The inclusion criteria of the studies were (1) populations: women of Asian ethnicity; (2) exposure: having a family history of breast cancer; (3) comparator: women without a family history of breast cancer; (4) outcomes: breast cancer; (5) study types: case–control or cohort study that reported risk ratios, odds ratios (ORs), or hazard ratios to assess family history as a risk factor of breast cancer; (6) time frame: published before 31 March 2023; and (7) full text of the articles are available. Conference abstracts, publications in a non-English language or studies of non-human research were excluded.

### Data extraction

After determining the included studies, the following information was extracted from each publication using an Excel form designed for this study: authors, publication date, study location, study design, information on cases and controls (e.g. sample size, recruitment methods, cases’ age at diagnosis, recruitment year, control and case type and matching technique), type of family history (i.e. relatedness and number of affected relatives and the relatives’ age at diagnosis) and risk estimates with corresponding 95% confidence intervals (95% CIs). The data extraction was conducted by one investigator (H.W.) and checked by another investigator (S.L.), and any disagreement was resolved by discussion and consensus.

For the studies eligible for inclusion, we adopted the Risk of Bias in Non-randomised Studies of Exposure (ROBINS-E) criteria [17] to evaluate their quality on seven domains of bias: confounding, participant selection, exposure classification, departures from intended exposures, missing data, outcome measurement and reported result selection. The assessments on individual domains were summarised into an overall risk of bias assessment for each study to generate three levels of risk of bias: high, moderate and low risk of bias. We only included studies with a low to moderate risk of bias in the meta-analysis.

### Meta-analysis

The meta-analysis was conducted using Stata version 16.0 (Stata Corporation). The results were presented as ORs with corresponding 95% confidence intervals. We fitted random-effect models to evaluate the association between family history and breast cancer, assuming varying effect sizes across the studies. Apart from investigating the overall association across all studies, we also investigated the association by the type of family history, age and menopausal status of the participants and study location. For age-specified risk comparison, we compared the breast cancer risk of women aged ≥ 50 years with those of women aged < 50 years. The cut-off of 50 years old was chosen for comparison with the results from previous studies [1–3]; it was also the most common cut-off reported in our included studies that investigated the age-specific risk. Tests for differences (*Z*-test) between subgroups were conducted only using studies that reported estimates for all subgroups; for subgroups including different studies, we examined the consistency between subgroups by checking the overlap between the 95% CIs of the subgroup risks.

The *I*^2^ statistic was adopted to quantify the heterogeneity across the included studies. We visually evaluated publication bias using funnel plots and statistically assessed the bias through Egger’s and Begg’s tests.

### Evaluation of the systematic review

Grading of Recommendations Assessment, Development and Evaluation (GRADE) was adopted to rate the quality of scientific evidence in our systematic review [18]. The assessment of the certainty of the evidence was categorised as very low, low, moderate or high, with the evaluation of five main criteria: risk of bias, imprecision, inconsistency, indirectness and publication bias.

## Results

### Literature search

**Fig. 1:**
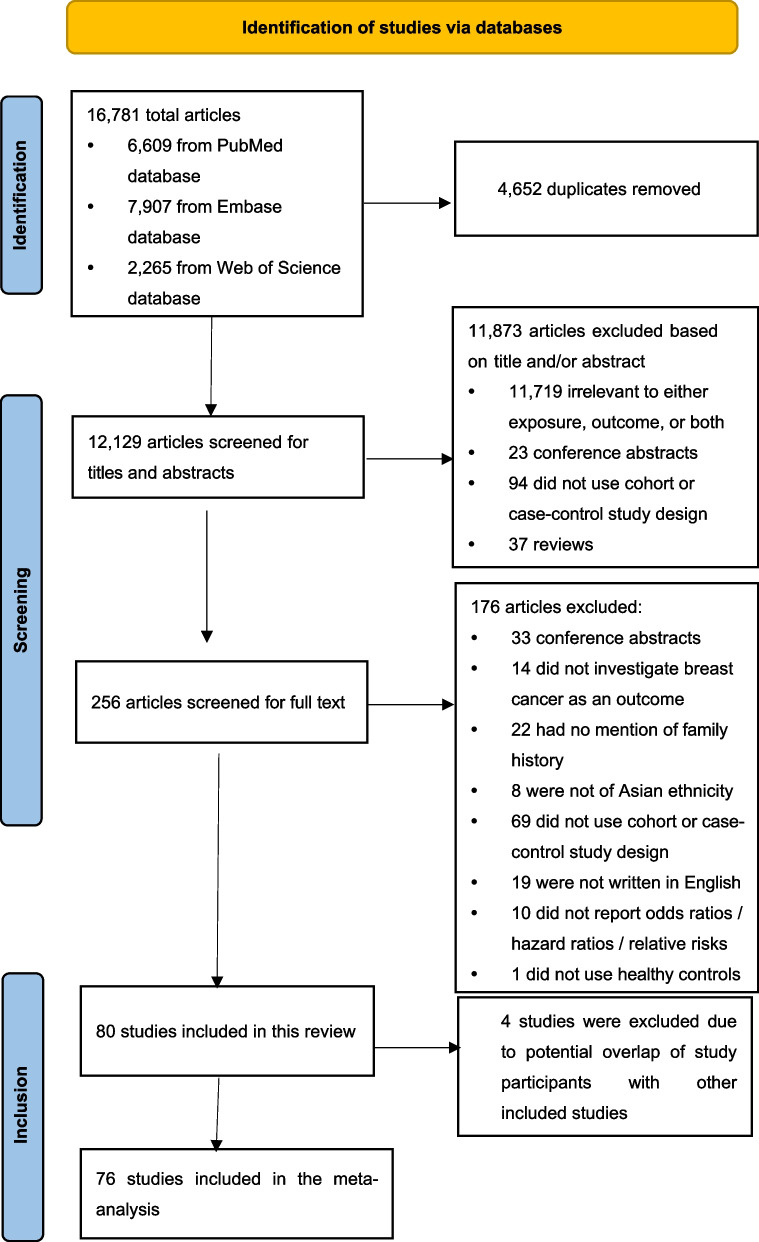
Flow diagram of the literature search and screening process

The search identified 16,781 articles; of these, 4,652 duplicates were removed, and another 12,129 articles were excluded after screening the titles and abstracts (Fig. 1). The remaining 256 were included for full-text screening, with 176 articles not meeting the inclusion criteria excluded. This process resulted in 80 studies eligible for inclusion in this systematic review [19–98]. We excluded four studies [95–98] for analysis since the participants in these studies were suspected to be overlapping with those of other studies from the same authors [54, 92]. We kept the latter studies that included more participants; therefore, 76 studies were included in the meta-analysis (Additional file 2: Table S1).

### Quality assessment

We found that all the studies had low to moderate levels of overall bias; therefore, we included all the studies in this meta-analysis. The individual quality assessment levels are presented in Additional file 2: Table S2.

The studies included five prospective cohort studies and 71 case–control studies in 25 countries and regions, published between 1984 and 2022. Studies in China (12 studies), Iran (11 studies), and Japan (8 studies) accounted for a large proportion of the included studies. There were also three studies from the USA and one from England that studied women of Asian ancestry. A total of 5,184,024 women were included in our analysis, with a mean age of 48.3 years old. A summary of all the included studies is presented in Additional file 2: Table S1.

### Family history in any relative

**Fig. 2:**
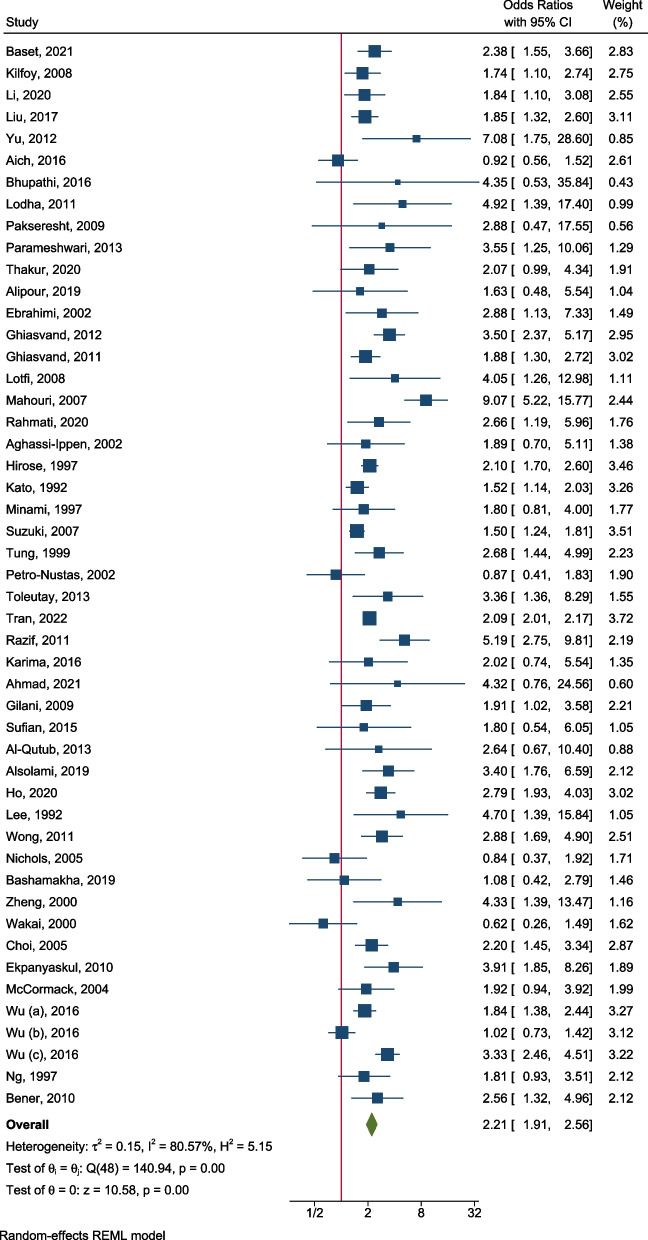
Risk of breast cancer for women with a family history in any relatives. *θ*_*i*_ (*i* = 1…49) is the study-specific effect size of the association on the log scale, *θ* is the pooled effect size of the association on the log scale, test of *θ*_*i*_ = *θ*_*j*_ is to test whether the effect sizes are homogenous across the studies, and test of *θ* = 0 is to test whether the pooled effect size equals to 0

Forty-nine studies including 48 case–control studies and one cohort study reported the risk of breast cancer associated with having a family history in any relative. The ORs ranged from 0.6 to 9.1, with 92% (45/49) of studies reporting an OR over 1. The pooled OR was 2.21 (95% CI: 1.91, 2.56) (Table 1; Fig. 2).

**Table 1:** Association between family history and breast cancer risk for Asian women

Type of family history	No. of studies	OR range	Pooled OR (95% CI)
Family history in any relatives	49	0.62–0.97	2.21 (1.91, 2.56)
By control type			
Hospital-based control	24	0.62–4.35	2.15 (1.84, 2.51)
Population-based control	24	0.84–7.08	2.24 (1.73, 2.89)
By the woman’s age			
Age < 50 years	2	1.81–3.33	1.87 (1.41, 2.48)
Age ≥ 50 years	2	1.13–2.04	1.97 (1.49, 2.60)
By menopausal status			
Pre-menopausal	3	1.61–5.33	2.84 (1.50, 5.37)
Post-menopausal	3	0.92–4.61	2.09 (0.92, 4.78)
By region			
East and Southeast Asia	23	0.62–7.08	2.05 (1.72, 2.43)
The rest of Asia	23	0.87–9.07	2.45 (1.90, 3.16)
Non-Asian countries	3	1.84–4.30	2.26 (1.42, 3.59)
Family history in first-degree relatives	33	0.50–9.52	2.46 (2.03, 2.97)
By menopausal status			
Pre-menopausal	3	2.21–5.33	3.21 (1.84, 5.61)
Post-menopausal	3	2.43–4.61	2.78 (1.60, 4.82)
By the type of relatives			
Mother	7	1.79–3.97	2.51 (1.88, 3.34)
Sister	7	1.23–5.80	2.03 (1.45, 2.83)
Family history in second-degree relatives	10	0.57–3.89	2.05 (1.56, 2.68)

Of the case–control studies, 24 studies selected controls from hospitals. The range of OR estimates was 0.62–4.35, with only one study reporting an OR smaller than 1, and the pooled OR was 2.15 (95% CI: 1.84, 2.51) (Table 1; Additional file 3: Fig. S1). The other 24 studies selected population-based controls. The range of OR estimates was relatively broad (0.84–7.08), with five studies reporting an OR close to the null; the pooled OR was 2.24 (95% CI: 1.73, 2.89) (Table 1; Additional file 3: Fig. S2).

Age-specific associations were reported in 17 studies. There were four studies that reported the association for women younger than 50 years old, with a pooled OR of 1.86 (95% CI: 1.47, 2.35) (Additional file 3: Fig. S3). Five studies reported the association for women older than 50 years old, with a pooled OR of 2.04 (95% CI: 1.27, 3.28) (Additional file 3: Fig. S4). Two studies reported results for both age groups; based on the results of the two studies, there was no evidence (ratio of the pooled ORs: 0.95, 95% CI: 0.64, 1.41, *P* = 0.8) that there was a difference for women under 50 years (pooled OR: 1.87, 95% CI: 1.41, 2.48) compared with those over 50 years (pooled OR: 1.97, 95% CI: 1.49, 2.60) (Table 1; Additional file 3: Fig. S5). Three studies reported ORs by menopausal status, and the pooled ORs for pre-menopausal (OR: 2.84, 95% CI 1.50–5.37) and post-menopausal (OR: 2.09, 95% CI 0.92–4.78) women were similar (ratio of the pooled ORs: 1.36, 95% CI: 0.48, 3.85, *P* = 0.56; Table 1; Additional file 3: Fig. S6).

Twenty-three studies reported the risk of breast cancer in East and Southeast Asia, with a pooled OR of 2.05 (95% CI: 1.72, 2.43) (Table 1; Additional file 3: Fig. S7). As for the studies in other regions of Asia, the pooled OR was 2.45 (95% CI: 1.90, 3.16) (Table 1; Additional file 3: Fig. S8). Three studies reported the risk for women of Asian ancestry living in non-Asian countries, with a pooled OR of 2.26 (95% CI: 1.42, 3.59) (Table 1; Additional file 3: Fig. S9), similar to the pooled OR of 2.18 (95% CI: 1.85, 2.58) for women living in Asian countries.

### Family history in first-degree relatives

**Fig. 3:**
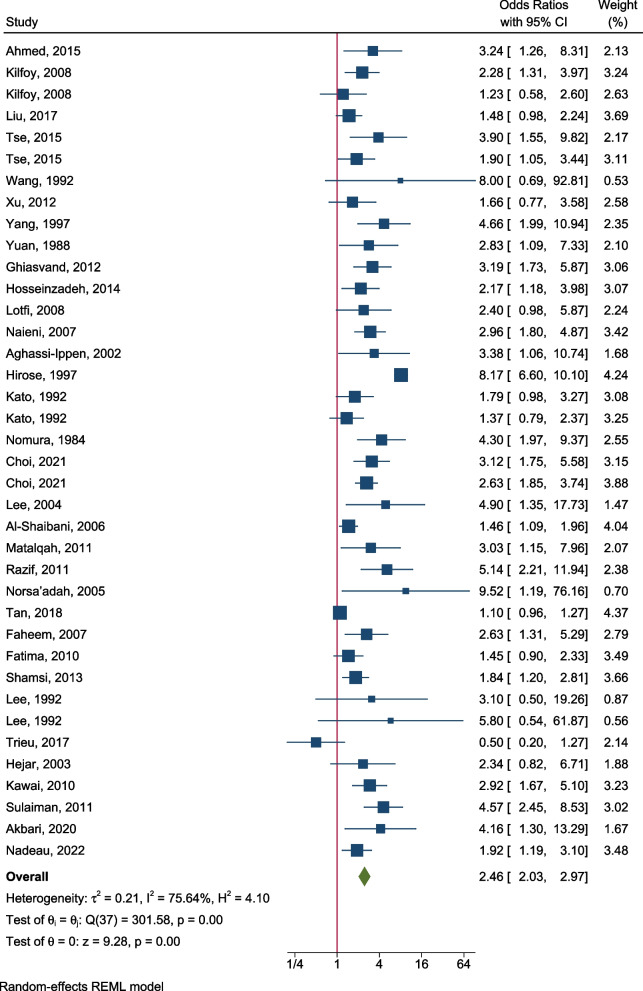
Risk of breast cancer for women with a family history in first-degree relatives. *θ*_*i*_ (*i* = 1…38) is the study-specific effect size of the association on the log scale, *θ* is the pooled effect size of the association on the log scale, test of *θ*_*i*_ = *θ*_*j*_ is to test whether the effect sizes are homogenous across the studies, and test of *θ* = 0 is to test whether the pooled effect size equals to 0

Thirty-three studies reported the risk of breast cancer for women with a family history in at least one first-degree relative (Fig. 3). The range of OR estimates was wide (0.5–9.5), and only one study reported an OR smaller than 1. The pooled OR was 2.46 (95% CI: 2.03, 2.97; Table 1). There was no evidence that the risks for pre-menopausal (OR: 3.21, 95% CI: 1.84, 5.61) and post-menopausal (OR: 2.78, 95% CI: 1.60, 4.82) women were different (ratio of the pooled ORs: 1.15, 95% CI: 0.53, 2.53, *P* = 0.72) (Table 1; Additional file 3: Fig. S10). Seven studies reported the association by the type of relative (Table 1; Additional file 3: Fig. S11): there was no evidence (ratio of the pooled ORs: 1.24, 95% CI: 0.80, 1.92, *P* = 0.35) that there was a difference in the association for women with a family history in mothers (OR: 2.51, 95% CI: 1.88, 3.34) and women with a family history in sisters (OR: 2.03, 95% CI: 1.45, 2.83).

### Family history in second-degree relatives

Ten studies reported the risk of breast cancer in women with a family history in at least one second-degree relative (Table 1; Additional file 3: Fig. S12). The ORs ranged from 0.57 to 3.89, with all but two studies reporting an OR greater than 1. The pooled OR estimate was 2.05 (95% CI: 1.56, 2.68).

### Publication bias

**Fig. 4:**
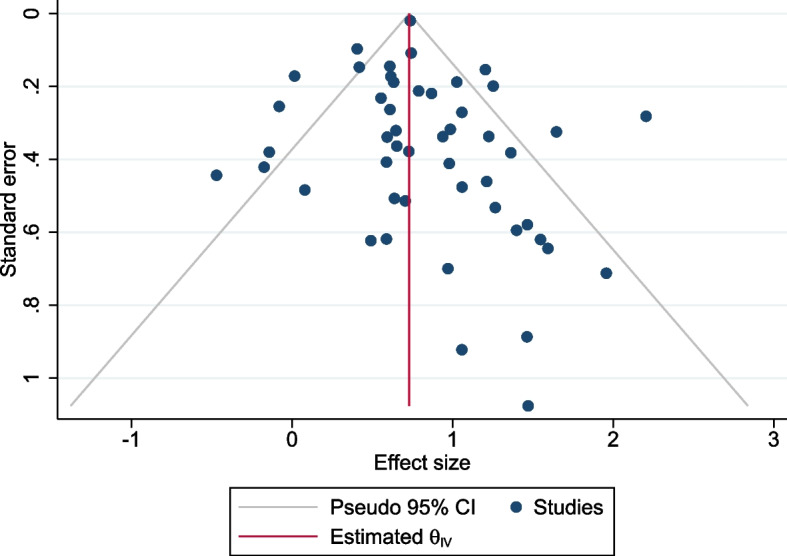
Funnel plot for studies reporting the risk associated with a family history in any relatives. *θ*_*iv*_ is the pooled effect size of the association on the log scale across the included studies

For the association with family history in any relative and breast cancer risk, no evidence of asymmetry was found in the funnel plot (Fig. 4). Egger’s test (*P* = 0.11) and Begg’s test (*P* = 0.11) also suggest there was no appreciable evidence for publication bias.

**Fig. 5:**
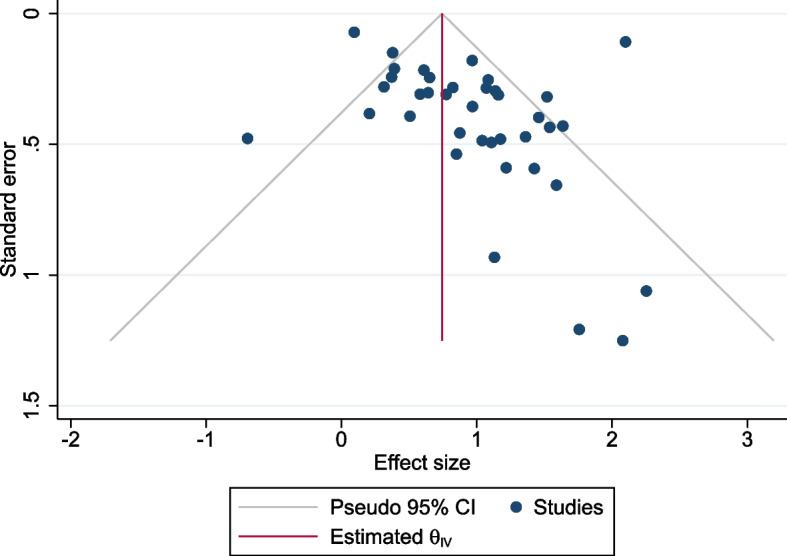
Funnel plot for studies reporting the risk associated with a family history in first-degree relatives. *θ*_*iv*_ is the pooled effect size of the association on the log-scale across the included studies

For the association with family history in first-degree relatives and breast cancer risk, the funnel plot showed an asymmetric pattern (Fig. 5). Egger’s test (*P* = 0.06) and Begg’s test (*P* = 0.04) also suggest there was some evidence for publication bias. Four sets of data in three studies [26, 70, 85] on the bottom right of the funnel plot appeared to drive the asymmetric pattern. We conducted a sensitivity analysis by removing these studies and found the pooled OR was 2.40 (95% CI: 1.98, 2.91) (Additional file 3: Fig. S13), similar to the 2.46 (95% CI: 2.03, 2.97) of the main analysis in Table 1, and no evidence of asymmetry was found (Additional file 3: Fig. S14; Egger’s test *P* = 0.25, Begg’s test *P* = 0.08). Therefore, the publication bias in the main analysis did not materially impact our estimate of the association with family history in first-degree relatives.

### GRADE assessment

This systematic review had moderate to high certainty according to the GRADE guidelines (Additional file 2: Table S3). All our included studies showed a moderate to low risk of bias with direct evidence to answer our research question. This was also ensured by relatively precise results and little publication bias.

## Discussion

This systematic review and meta-analysis included 76 studies published between 1984 and 2022 that reported the relationship between family history and breast cancer for Asian women. This review shows that Asian women with a family history of breast cancer have a higher risk of breast cancer than those who do not have a family history: the familial relative risk associated with an affected first-degree relative was around twofold. The observed magnitude of familial risk for Asian women is similar to those observed for women of European ancestry [1–3]. Such consistency implies that familial factors implicated in breast cancer might be similar for Asian and European ancestry women. The familial factors include genetic factors such as rare germline mutations in known susceptibility genes and common genetic variants [9]. We did not observe a difference in the familial risk between Asian women living in Asia and those living in non-Asian countries, which supports the role of genetic factors in causing the familial risk. This is also supported by our finding that the familial risk was similar across Asian regions with distinct living environments and cultures. There is also evidence for non-genetic factors explaining part of the familial risk, especially at a younger age, as suggested by the Nordic Twin Study [10].

People of non-European ancestry are currently underrepresented in genetic research of breast cancer, such as that genome-wide association studies are predominately for women of European ancestry, and there is a lack of data available to interpret the pathogenicity of variants in known breast cancer predisposition genes in non-Europeans. As a result, findings from some breast cancer genetic research might not be applicable to people of non-European ancestry. For example, the breast cancer polygenic risk score developed for women of European ancestry has a smaller effect size for Asian women, implying that the score does not predict breast cancer risk for Asian women as well as for European women [99]. Given that ~ 200 genetic loci have been found to explain breast cancer familial risk for European women whilst only a few for Asian women [100–103], our finding that Asian and European women have similar magnitudes of breast cancer familial risk implies that there are more breast cancer genetic loci yet to be found for Asian women and the risk associated with known predisposition genes yet to be determined. Therefore, our finding could be informative for future studies to identify breast cancer genetic variants for Asian women and for breast cancer genetic research for both aetiology and risk prediction that uses the multi-ancestry approach [103].

We tested for differences between the subgroups reported in the same studies but did not find any evidence that the familial risk differed by the woman’s age, menopausal status, geographical region and type of relative or control type. Some of the findings were inconsistent with the results for women of European ancestry where the risk association decreases with familial relatedness and the woman’s age [1–3]. This inconsistency could be due to a low number of Asian studies reporting the estimates for these categories. There were only 10 studies about the association with family history in second-degree relatives and 7 studies that reported age-specific risk by age of 50 years. Furthermore, we could only use two studies to test the difference in the risk by age of 50 years; such a low number of studies could have impacted our statistical power to find a difference.

The insufficient number of available studies also limited our ability to provide precise estimates for the risks associated with an affected mother or an affected sister, nor to detect the difference between the two types of first-degree relatives. Studies for women of European ancestry found that the risk associated with an affected sister is somewhat greater than the risk associated with an affected mother, especially for women younger than 50 years old: the OR is 2.41 (95% CI 1.86, 3.12) for having an affected mother and 3.18 (95% CI 2.15, 4.72) for having an affected sister [3]. A higher risk associated with affected sisters is consistent with that recessively inherited genes or variants are implicated in breast cancer risk [9]. Our study suggests that more research is needed to better understand the variation in risk associated with the type of relatives for Asian women.

A familial relative risk of two does not necessarily imply that every Asian woman with a family history should participate in cancer screening. However, women who have a strong family history should consider more frequent screenings [104, 105]. Albright et al. [2], for example, found that women with more than five first-degree relatives have a fivefold increased risk; however, we have no information on such number of affected relatives in our included studies. Screening programme participation rates in Asia are low, and knowledge, culture, attitude and feeling, and economic and logistical barriers were suggested to be the reasons [12, 106]. These potential barriers could be addressed by government subsidy plans, increasing awareness and being culturally sensitive when managing Asian families.

Family history, especially multi-generational one, is included as an important predictor in the risk models that are commonly used for women of European ancestry [4–7]. For Asian women, the Asian American Breast Cancer Study model has been shown to accurately predict risk for Asian Americans [107]; however, to the best of our knowledge, for women living in Asian countries, there are no widely used breast cancer risk models that consider family history.

Our study has several strengths, including searching published literature in three databases, using three search strategies to minimise the potential omission of eligible studies, quality assessment of the included studies, investigating breast cancer familial risk between several subgroups, and our results having moderate to high certainty according to the GRADE guidelines.

Nevertheless, in addition to the limitations mentioned above, other limitations need to be considered when interpreting our results. First, we defined Asian women as those who live or have origin in Asian countries in the United Nations geoscheme, but populations in these countries are not homogenous, especially in genetic ancestry. Second, although the included studies were from 25 countries and regions which is more than previous reviews [13, 14], due to the availability of literature, the included studies were not from every Asian country; therefore, our findings might not be applicable to the whole Asian population. Third, we only found four studies of Asian women in non-Asian countries, which might limit our ability to find a difference in the familial risk between them and those living in Asian countries. Fourth, recall bias, especially in case–control studies, could bias the findings of our included studies. Women with breast cancer might be more likely to recall having a family history than controls, which could bias the results away from null to overestimate the effect. On the other hand, it is possible that some women underreported their family history in either the case or control groups due to social and cultural reasons in Asia [108]. Validation of reported family history could reduce the recall bias and misclassification of the family history; however, this is expensive and time-consuming and thus might not be practical.

## Conclusions

This systematic review and meta-analysis provided evidence that family history is associated with an increased risk of breast cancer for Asian women, and the familial risk appears to be similar to those observed for women of European ancestry, suggesting there are similar familial factors implicated in breast cancer risk across ancestries. Genetic risk factors play a substantial role in explaining the familial risk, as similar familial risks were observed across different countries, living environments and cultures.

## Supplementary Information

**Additional file 1.**

**Additional file 2.**

**Additional file 3.**

## Data Availability

This study used the summary statistics of published studies. All data are available in the included studies.

## Abbreviations

BCRAT: Breast Cancer Risk Assessment Tool
BOADICEA: Breast and Ovarian Analysis of Disease Incidence and Carrier Estimation Algorithm model
CI: Confidence interval
GRADE: Grading of Recommendations Assessment, Development and Evaluation
IBIS: International Breast Cancer Intervention Study model
OR: Odds ratio
PRISMA: Preferred Reporting Items for Systematic Reviews and Meta-analysis Statement
ROBINS-E: Risk of Bias in Non-randomised Studies of Exposure
