# Supplementary material for: Family history and breast cancer risk for Asian women: a systematic review and meta-analysis
Source: BMC Med. 2023 Jul 3;21:239. doi: 10.1186/s12916-023-02950-3 (PMC10318753; doi:10.1186/s12916-023-02950-3)
Supplement: Supplementary file 1 — Additional file 1. [file 12916_2023_2950_MOESM1_ESM.docx]

## Literature search methods

PubMed, Embase, and Web of Science were searched for this systematic review using three methods. The search was initially conducted on February 1^st^ 2021, and another research was conducted on April 19^th^ 2023 during the revision of the manuscript. Details of the search strings for the three databases are provided below.

### Method 1

- Database 1: PubMed (2,695 results)

("family history" OR "family" OR "familial") AND ("breast cancer" OR "Breast Neoplasms"[Mesh] OR "breast") AND (Asia [Mesh])

Filters applied: Humans, English.

- Database 2: Embase (3,666 results)

('family history'/exp OR 'family history' OR 'family' OR 'familial') AND ('breast cancer'/exp OR 'breast cancer' OR 'breast') AND 'asia'/exp AND 'human'/de

- Database 3: Web of Science (1,360 results)

TS=(“Breast Neoplasms” OR “Breast Neoplasm” OR “Neoplasm, Breast” OR “Breast Tumors” OR “Breast Tumor” OR “Tumor, Breast” OR “Tumors, Breast” OR “Neoplasms, Breast” OR “Breast Cancer” OR “Cancer, Breast” OR “Mammary Cancer” OR “Cancer, Mammary” OR “Cancers, Mammary” OR “Mammary Cancers” OR “Malignant Neoplasm of Breast” OR “Breast Malignant Neoplasm” OR “Breast Malignant Neoplasms” OR “Malignant Tumor of Breast” OR “Breast Malignant Tumor” OR “Breast Malignant Tumors” OR “Cancer of Breast” OR “Cancer of the Breast” OR “Mammary Carcinoma, Human” OR “Carcinoma, Human Mammary” OR “Carcinomas, Human Mammary” OR “Human Mammary Carcinomas” OR “Mammary Carcinomas, Human” OR “Human Mammary Carcinoma” OR “Mammary Neoplasms, Human” OR “Human Mammary Neoplasm” OR “Human Mammary Neoplasms” OR “Neoplasm, Human Mammary” OR “Neoplasms, Human Mammary” OR “Mammary Neoplasm, Human” OR “Breast Carcinoma” OR “Breast Carcinomas” OR “Carcinoma, Breast” OR “Carcinomas, Breast”) AND TS=(“family history” OR family OR familial ) AND TS=(“Asia” OR “Asia, Central” OR “Asia, Northern” OR “Asia, Southeastern” OR “Asia, Western” OR “Far East” OR Kazakhstan OR Kyrgyzstan OR Tajikistan OR Turkmenistan OR Uzbekistan OR Russia OR Borneo OR Brunei OR Cambodia OR Indochina OR Indonesia OR Laos OR Malaysia OR Mekong Valley OR Myanmar OR Philippines OR Singapore OR Thailand OR Timor-Leste OR Vietnam OR Bangladesh OR Bhutan OR India OR Middle East OR Nepal OR Pakistan OR Sri Lanka OR China OR Japan OR Korea OR Mongolia OR Taiwan) AND LA=English

### Method 2

- Database 1: PubMed (1,804 results)

(("Asia"[Mesh]) AND ("Risk Factors"[Mesh])) AND ("Breast Neoplasms"[Mesh])

Filters applied: Humans, English

- Database 2: Embase (3,101 results)

'risk factor'/exp AND ('breast cancer'/exp OR 'breast cancer' OR 'breast') AND 'asia'/exp AND 'human'/de

- Database 3: Web of Science (440 results)

TS=(“Breast Neoplasms” OR “Breast Neoplasm” OR “Neoplasm, Breast” OR “Breast Tumors” OR “Breast Tumor” OR “Tumor, Breast” OR “Tumors, Breast” OR “Neoplasms, Breast” OR “Breast Cancer” OR “Cancer, Breast” OR “Mammary Cancer” OR “Cancer, Mammary” OR “Cancers, Mammary” OR “Mammary Cancers” OR “Malignant Neoplasm of Breast” OR “Breast Malignant Neoplasm” OR “Breast Malignant Neoplasms” OR “Malignant Tumor of Breast” OR “Breast Malignant Tumor” OR “Breast Malignant Tumors” OR “Cancer of Breast” OR “Cancer of the Breast” OR “Mammary Carcinoma, Human” OR “Carcinoma, Human Mammary” OR “Carcinomas, Human Mammary” OR “Human Mammary Carcinomas” OR “Mammary Carcinomas, Human” OR “Human Mammary Carcinoma” OR “Mammary Neoplasms, Human” OR “Human Mammary Neoplasm” OR “Human Mammary Neoplasms” OR “Neoplasm, Human Mammary” OR “Neoplasms, Human Mammary” OR “Mammary Neoplasm, Human” OR “Breast Carcinoma” OR “Breast Carcinomas” OR “Carcinoma, Breast” OR “Carcinomas, Breast”) AND TS=(“risk factor”) AND TS=(“Asia” OR “Asia, Central” OR “Asia, Northern” OR “Asia, Southeastern” OR “Asia, Western” OR “Far East” OR Kazakhstan OR Kyrgyzstan OR Tajikistan OR Turkmenistan OR Uzbekistan OR Russia OR Borneo OR Brunei OR Cambodia OR Indochina OR Indonesia OR Laos OR Malaysia OR Mekong Valley OR Myanmar OR Philippines OR Singapore OR Thailand OR Timor-Leste OR Vietnam OR Bangladesh OR Bhutan OR India OR Middle East OR Nepal OR Pakistan OR Sri Lanka OR China OR Japan OR Korea OR Mongolia OR Taiwan) AND LA=English

### Method 3

- Database 1: PubMed (2,110 results)

(("Breast Neoplasms"[Mesh]) AND ("Risk Factors"[Mesh])) AND (race OR ethnicity)

Filters applied: Humans, English

- Database 2: Embase (1,140 results)

'risk factor'/exp AND ('breast cancer'/exp OR 'breast cancer' OR 'breast') AND ('race' OR ‘ethnicity)/exp AND 'human'/de

- Database 3: Web of Science (440 results)

TS=(“Breast Neoplasms” OR “Breast Neoplasm” OR “Neoplasm, Breast” OR “Breast Tumors” OR “Breast Tumor” OR “Tumor, Breast” OR “Tumors, Breast” OR “Neoplasms, Breast” OR “Breast Cancer” OR “Cancer, Breast” OR “Mammary Cancer” OR “Cancer, Mammary” OR “Cancers, Mammary” OR “Mammary Cancers” OR “Malignant Neoplasm of Breast” OR “Breast Malignant Neoplasm” OR “Breast Malignant Neoplasms” OR “Malignant Tumor of Breast” OR “Breast Malignant Tumor” OR “Breast Malignant Tumors” OR “Cancer of Breast” OR “Cancer of the Breast” OR “Mammary Carcinoma, Human” OR “Carcinoma, Human Mammary” OR “Carcinomas, Human Mammary” OR “Human Mammary Carcinomas” OR “Mammary Carcinomas, Human” OR “Human Mammary Carcinoma” OR “Mammary Neoplasms, Human” OR “Human Mammary Neoplasm” OR “Human Mammary Neoplasms” OR “Neoplasm, Human Mammary” OR “Neoplasms, Human Mammary” OR “Mammary Neoplasm, Human” OR “Breast Carcinoma” OR “Breast Carcinomas” OR “Carcinoma, Breast” OR “Carcinomas, Breast”) AND TS=(“risk factor”) AND TS=(race OR ethnicity) AND LA=English
