# Supplementary material for: Family history and breast cancer risk for Asian women: a systematic review and meta-analysis
Source: BMC Med. 2023 Jul 3;21:239. doi: 10.1186/s12916-023-02950-3 (PMC10318753; doi:10.1186/s12916-023-02950-3)
Supplement: Supplementary file 2 — Additional file 2. [file 12916_2023_2950_MOESM2_ESM.docx]

**Table S1** Summary of the included studies

| **Study** | **Published Year** | **Location** | **Study Type** | | **Recruited Year** | **Age range** | **No. of Cases** | **No. of Controls** | **Control Type** |
| --- | --- | --- | --- | --- | --- | --- | --- | --- | --- |
| Baset et al. [19] | 2021 | Afghanistan | case-control study | 2018-2019 | | >=30 | 201 | 201 | hospital |
| Ahmed et al. [20] | 2015 | Bangladesh | case-control study | 2011-2014 | | 25-75 | 80 | 80 | hospital |
| Kilfoy et al. [21] | 2008 | China | prospective cohort study | 1996-2000 | | 40-70 | 570 | 72652 |  |
| Li et al. [22] | 2020 | China | case-control study | 2015-1029 | | 20-84 | 495 | 478 | hospital |
| Li et al. [23] | 2005 | China | case-control study | 1989-1991 | | Not reported | 384 | 367 | population |
| Liu et al. [24] | 2017 | China | case-control study | 2012-2013 | | 25-70 | 1498 | 1498 | hospital |
| Qiu et al. [25] | 2012 | China | case-control study | 2007-2009 | | 23-80 | 500 | 500 | population |
| Wang et al. [26] | 1992 | China | case-control study | 1985-1986 | | 20-55 | 300 | 300 | population |
| Xu et al. [27] | 2012 | China | case-control study | 2008 | | 30-65 | 416 | 1156 | population |
| Yu et al. [28] | 2012 | China | case-control study | Not reported | | 25-70 | 103 | 309 | population |
| Yuan et al. [29] | 1988 | China | case-control study | 1984-1985 | | 20-69 | 534 | 534 | population |
| Zheng et al. [30] | 2000 | China | case-control study | 1997-1999 | | 20-80 | 404 | 404 | hospital |
| Tse et al. [31] | 2015 | Hong Kong, China | case-control study | 2011-2014 | | 20-84 | 747 | 781 | Hospital |
| Yang et al. [32] | 1997 | Taiwan, China | case-control study | 1993-1994 | | 20-80 | 244 | 450 | hospital |
| McCormack et al. [33] | 2004 | England (South Asian^^[[1]](#footnote-1)^^) | case-control study | 1995-1999 | | <75 | 240 | 477 | population |
| Aich et al. [34] | 2016 | India | case-control study | 2008-2012 | | Not reported | 1463 | 1440 | population |
| Bhupathi et al. [35] | 2016 | India | case-control study | Not reported | | 47.32 | 69 | 30 | Not reported |
| Lodha et al. [36] | 2011 | India | case-control study | 2008-2009 | | 28-78 | 215 | 215 | population |
| Pakseresht et al. [37] | 2009 | India | case-control study | 2006 | | 24-80 | 115 | 217 | hospital |
| Parameshwari et al. [38] | 2013 | India | case-control study | 2012 | | 32-70 | 20 | 80 | population |
| Thakur et al. [39] | 2020 | India | case-control study | 2014 | | Not reported | 377 | 346 | hospital |
| Wakai et al. [40] | 2000 | Indonesia | case-control study | 1992-1995 | | 20-79 | 226 | 452 | hospital |
| Alipour et al. [41] | 2019 | Iran | case-control study | 2004-2008 | | 40-75 | 124 | 400 | population |
| Dianatinasab et al. [42] | 2019 | Iran | case-control study | 2014-2017 | | Not reported | 1009 | 1009 | hospital |
| Ebrahimi et al. [43] | 2002 | Iran | case-control study | 1997-1998 | | 32-81 | 286 | 249 | hospital |
| Ghiasvand et al. [44] | 2012 | Iran | case-control study | 2005-2008 | | 50-89 | 493 | 493 | hospital |
| Ghiasvand et al. [45] | 2011 | Iran | case-control study | 2005-2008 | | 20-50 | 521 | 521 | hospital |
| Hosseinzadeh et al. [46] | 2014 | Iran | case-control study | 2012-2013 | | Not reported | 140 | 280 | hospital |
| Lotfi et al. [47] | 2008 | Iran | case-control study | 2006 | | 31-75 | 80 | 80 | population |
| Mahouri et al. [48] | 2007 | Iran | case-control study | 2000-2002 | | 48.45 | 168 | 504 | population |
| Naieni et al. [49] | 2007 | Iran | case-control study | 2004 | | 19-80 | 250 | 500 | population |
| Rahmati et al. [50] | 2020 | Iran | case-control study | 2015-2016 | | 45.45 | 50 | 50 | hospital |
| Akbari et al. [51] | 2020 | Iran | case-control study | 2014-2017 | | 25-55 | 732 | 584 | hospital |
| Aghassi-Ippen et al. [52] | 2002 | Israel | case-control study | 1990-1998 | | 30-70 | 72 | 140 | hospital |
| Chaudary et al. [53] | 1991 | Japan | case-control study | 1979-1980 | | 47.72 | 200 | 335 | population |
| Hirose et al. [54] | 1997 | Japan | case-control study | 1988-1995 | | 45.07 | 1551 | 28450 | hospital |
| Kato et al. [55] | 1992 | Japan | case-control study | 1990-1991 | | Not reported | 908 | 908 | hospital |
| Minami et al. [56] | 1997 | Japan | case-control study | 1987-1991 | | Not reported | 204 | 810 | population |
| Suzuki et al. [57] | 2007 | Japan | case-control study | 1988-2004 | | 20-79 | 3861 | 3861 | hospital |
| Tung et al. [58] | 1999 | Japan | case-control study | 1990-1995 | | 53.15 | 376 | 430 | hospital |
| Waka et al. [59] | 1995 | Japan | case-control study | 1990-1991 | | 20-80 | 300 | 900 | hospital |
| Kawai et al. [60] | 2010 | Japan | prospective cohort study | 1990-2003 | | 40-64 | 285 | 23779 |  |
| Petro-Nustas et al. [61] | 2002 | Jordan | case-control study | 1996 | | Not reported | 100 | 100 | population |
| Toleutay et al. [62] | 2013 | Kazakhstan | case-control study | 2011 | | 30-75 | 114 | 196 | population |
| Lee et al. [63] | 2004 | Korea | case-control study | Not reported | | 20- | 384 | 166 | hospital |
| Choi et al. [64] | 2005 | Korea | case-control study | 1995-2003 | | 47.70 | 1011 | 1011 | hospital |
| Choi et al. (2) [65] | 2021 | Korea | prospective cohort study | 2001-2013 | | Not reported | 981 | 128393 |  |
| Mai Tran et al. [66] | 2022 | Korea | prospective cohort study | 2009-2010 | | 40- | 77238 | 4758269 |  |
| Al-Shaibani et al. [67] | 2006 | Kuwait | case-control study | 2003-2004 | | 30-65 | 514 | 556 | hospital |
| Matalqah et al. [68] | 2011 | Malaysia | case-control study | 2009-2010 | | 22-83 | 150 | 150 | hospital |
| Mohd Razif et al. [69] | 2011 | Malaysia | case-control study | 2006-2007 | | 18-55 | 216 | 216 | population |
| Norsa'adah et al. [70] | 2005 | Malaysia | case-control study | 2000-2001 | | 28-70 | 147 | 147 | hospital |
| Tan et al. [71] | 2018 | Malaysia | case-control study | 2002-2016 | | 40-74 | 3683 | 3980 | hospital |
| Karim et al. [72] | 2016 | Malaysia | case-control study | 2007-2015 | | 31-92 | Not reported | Not reported | population |
| Hejar et al. [73] | 2003 | Malaysia | case-control study | 2001 | | 20-55 | 89 | 85 | hospital |
| Sulaiman et al. [74] | 2011 | Malaysia | case-control study | 2006-2007 | | 18-80 | 382 | 382 | population |
| Ahmad et al. [75] | 2021 | Pakistan | case-control study | 2017-2018 | | 26-80 | 163 | 163 | hospital |
| Faheem et al. [76] | 2007 | Pakistan | case-control study | 2005 | | 42.35 | 150 | 150 | population |
| Fatima et al. [77] | 2010 | Pakistan | prospective cohort study | 2009 | | 39±15 | 181 | 858 |  |
| Gilani et al. [78] | 2009 | Pakistan | case-control study | 1997-1998 | | 20-44 | 498 | 996 | population |
| Shamsi et al. [79] | 2013 | Pakistan | case-control study | 2009-2010 | | Not reported | 257 | 514 | hospital |
| Sufian et al. [80] | 2015 | Pakistan | case-control study | Not reported | | 30-80 | 108 | 108 | hospital |
| Bener et al. [81] | 2010 | Qatar | case-control study | 2008-2009 | | 22-84 | 167 | 341 | population |
| Al-Qutub et al. [82] | 2013 | Saudi Arabia | case-control study | 2010-2011 | | 19-50 | 151 | 166 | hospital |
| Alsolami et al. [83] | 2019 | Saudi Arabia | case-control study | 2014-2016 | | 45-75 | 214 | 218 | hospital |
| Ho et al. [84] | 2020 | Singapore | case-control study | 1994-1997 | | 50-64 | 474 | 27656 | population |
| Lee et al. [85] | 1992 | Singapore | case-control study | Not reported | | Not reported | 200 | 420 | hospital |
| Wong et al. [86] | 2011 | Singapore | case-control study | 1994-1997 | | 45-69 | 491 | 982 | population |
| Ng et al. [87] | 1997 | Singapore | case-control study | 1994-1996 | | 45-69 | 204 | 882 | population |
| Ekpanyaskul et al. [88] | 2010 | Thailand | case-control study | 2002-2004 | | 17-79 | 516 | 516 | hospital |
| Nomura et al. [89] | 1984 | U.S. (Japanese in Hawaii) | case-control study | 1975-1980 | | 45-74 | 183 | 183 | population |
| Wu et al. [90] | 2016 | U.S. (Chinese in the U.S.) | case-control study | 1995-2001 or 2003-2008 | | 25-74 | 891 | 896 | population |
|  |  | U.S. (Filipina in the U.S.) | case-control study | 1995-2001 or 2003-2006 | | 25-74 | 780 | 582 | population |
|  |  | U.S. (Japanese in the U.S.) | case-control study | 1995-2001 or 2003-2007 | | 25-74 | 519 | 505 | population |
| Nadeau et al. [91] | 2022 | U.S.(Indian in the U.S.) | case-control study | 1990-2016 | | 29-88 | 141 | 278 | hospital |
| Trieu et al. [92] | 2017 | Vietnam | case-control study | 2015 | | 48.94 | 269 | 519 | hospital |
| Nichols et al. [93] | 2005 | Vietnam & China | case-control study | 1993-1999 | | 20-57 | 682 | 649 | population |
| Bashamakha et al. [94] | 2019 | Yemen | case-control study | 2011-2015 | | Not reported | 105 | 210 | population |

The reference numbers are the same as those in the main text.


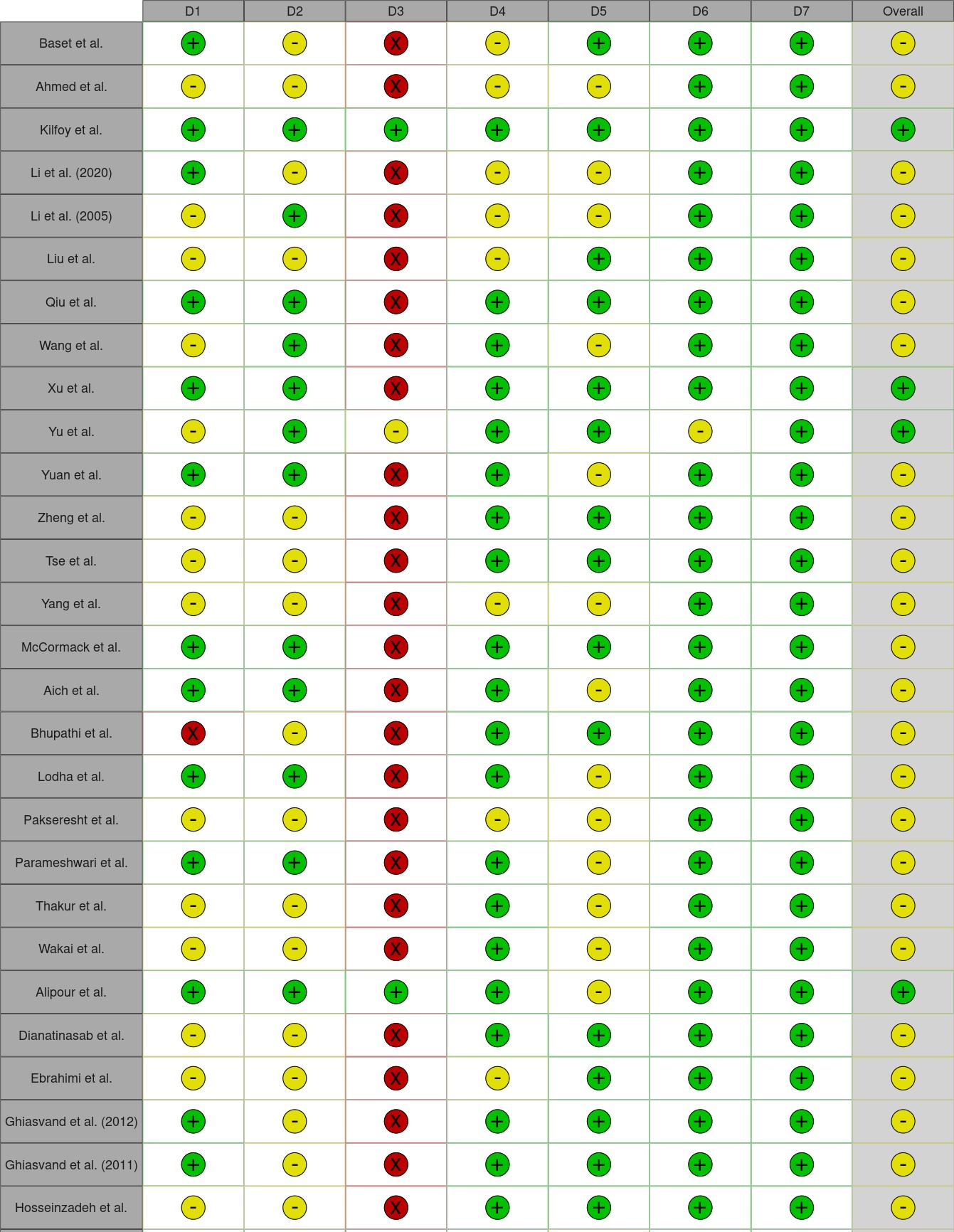
**Table S2** Quality Assessment by the ROBINS-E tool

Continued:


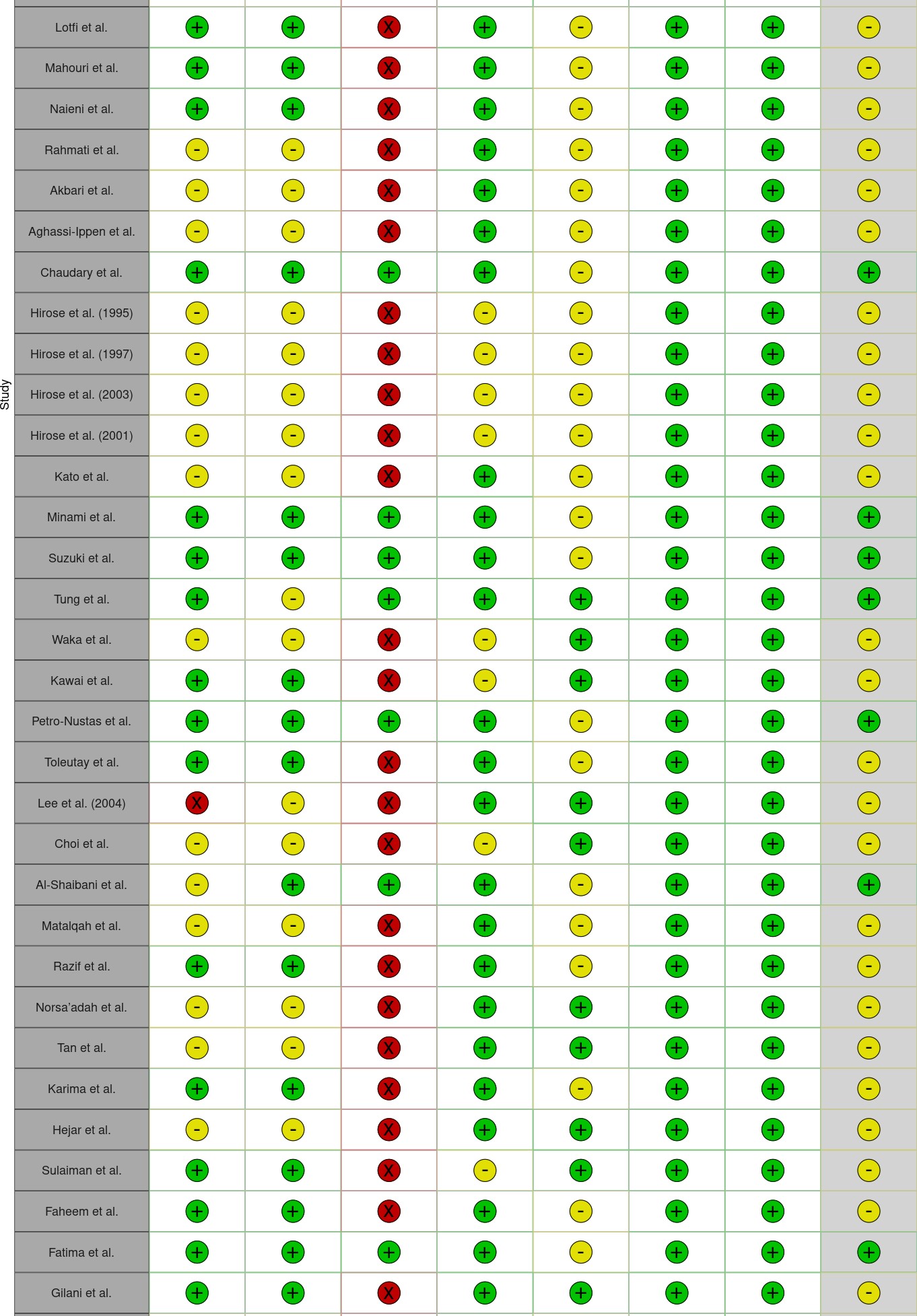


Continued:


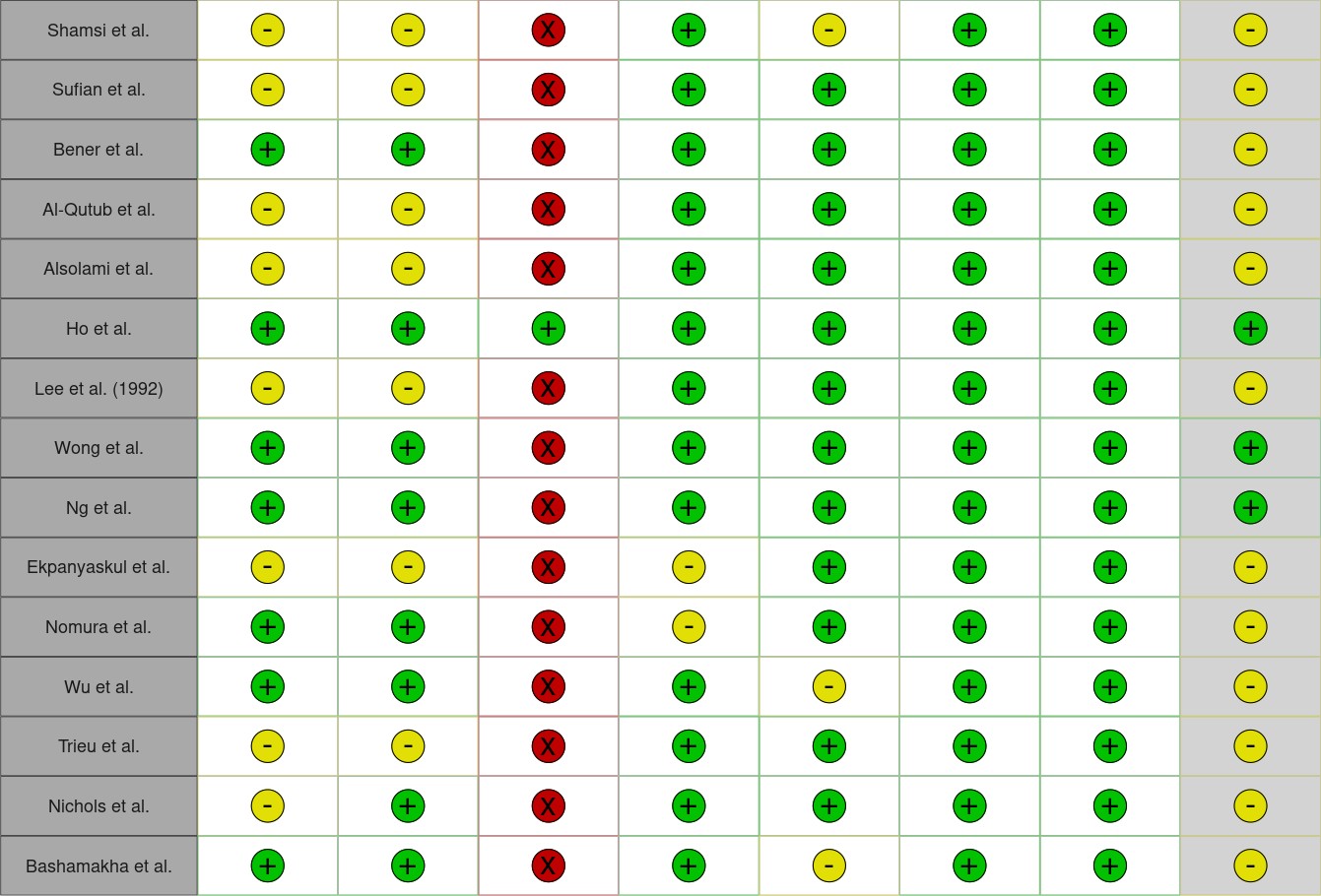

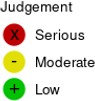


D1: Bias due to confounding,

D2: Bias due to selection of participants, D3: Bias in classification of interventions,

D4: Bias due to deviations from intended interventions, D5: Bias due to missing data,

D6: Bias in measurement of outcomes,

D7: Bias in selection of the reported result.

**Table S3** Assessment of certainty level by the GRADE guidelines

| **ITEM** | **EVALUATION** | **NOTES** | **RESULTS** |
| --- | --- | --- | --- |
| **Risk of bias** | Moderate to low risk of bias | Mainly from potential recall bias and misclassification of exposure | Certainty Upgraded |
| **Inconsistency** | High heterogeneity | I2= 80.6% (random-effect model) | Certainty Downgraded |
| **Indirectness** | Direct evidence | - Population (Asian women) were what we are interested in, and in an extensive range - The exposure and outcome were well- defined and able to answer our research question | Certainty Upgraded |
| **Imprecision** | Precision | The results were precise with narrow 95% CIs (e.g., the overall pooled OR: 2.21, 95%CI: 1.91, 2.56) | Certainty Upgraded |
| **Publication Bias** | No evidence | Symmetric funnel plot | Certainty Upgraded |

1. South Asian including Gujarati Hindu, Punjabi Hindu, Punjabi Sikh, Pakistani/Indian Muslim, and Bangladeshi Muslim in England [↑](#footnote-ref-1)
