# Supplementary material for: Family history and breast cancer risk for Asian women: a systematic review and meta-analysis
Source: BMC Med. 2023 Jul 3;21:239. doi: 10.1186/s12916-023-02950-3 (PMC10318753; doi:10.1186/s12916-023-02950-3)
Supplement: Supplementary file 3 — Additional file 3. [file 12916_2023_2950_MOESM3_ESM.docx]

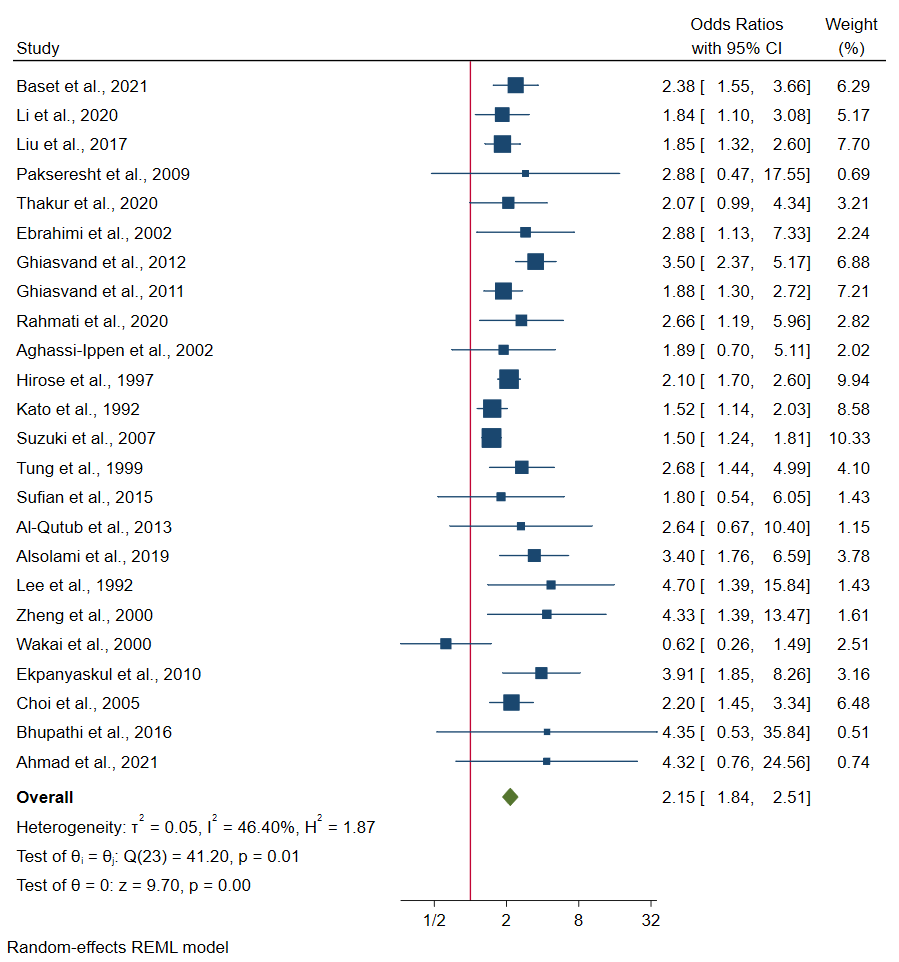


**Figure S1** Breast cancer risk for women with a family history in any relative from the studies with hospital-based controls


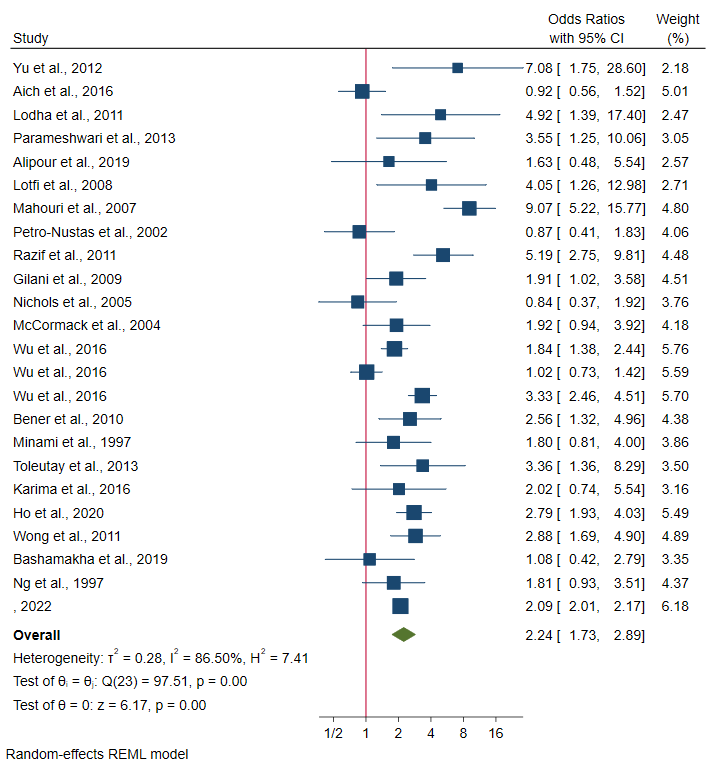


**Figure S2** Breast cancer risk for women with a family history in any relative from studies with population-based controls


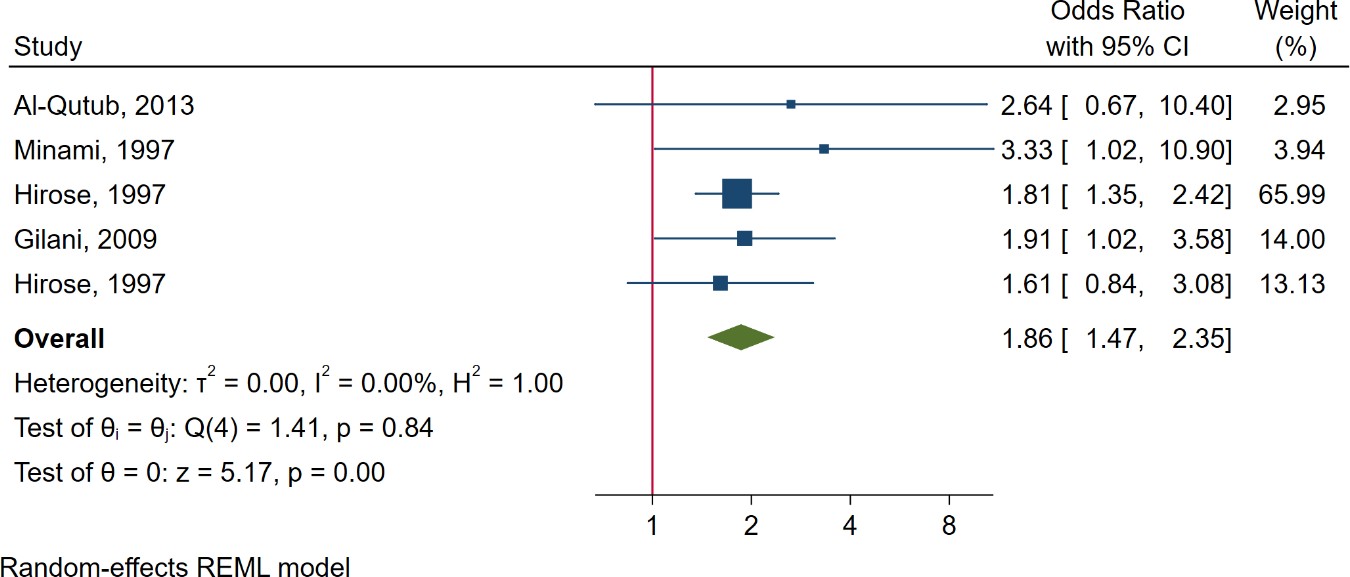


**Figure S3** Breast cancer risk for women aged younger than 50 years with a family history in any relative


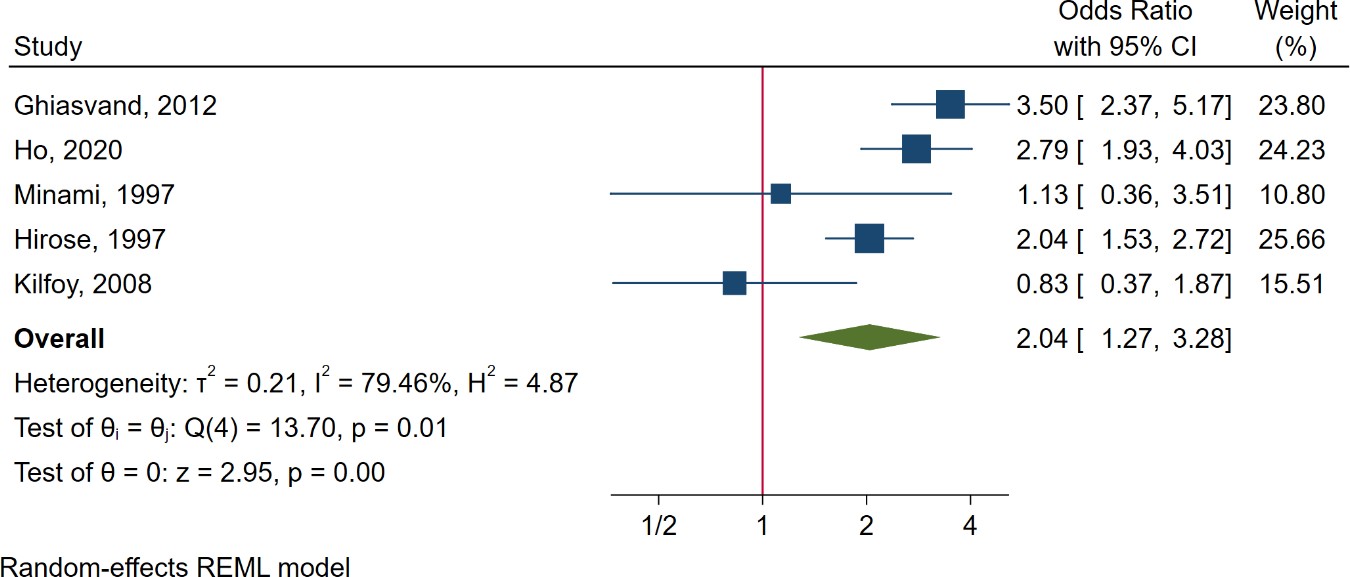


**Figure S4** Breast cancer risk for women aged older than 50 years with a family history in any relative


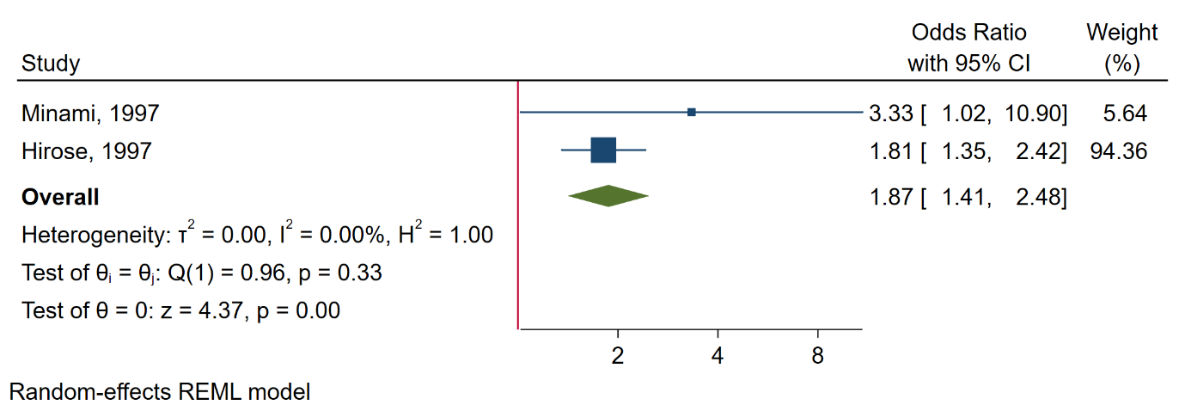

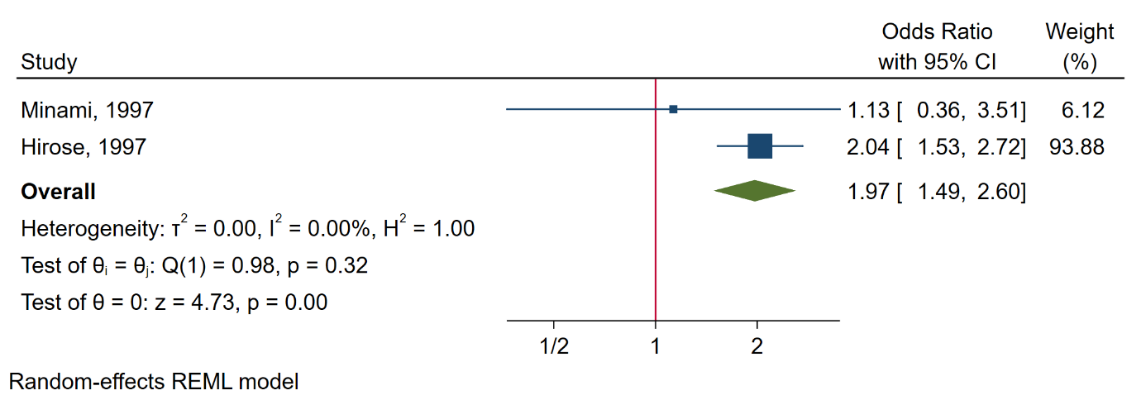


**Figure S5** Breast cancer risk comparison for women aged <50 years (top figure) and >50 years (bottom figure) with a family history in any relative


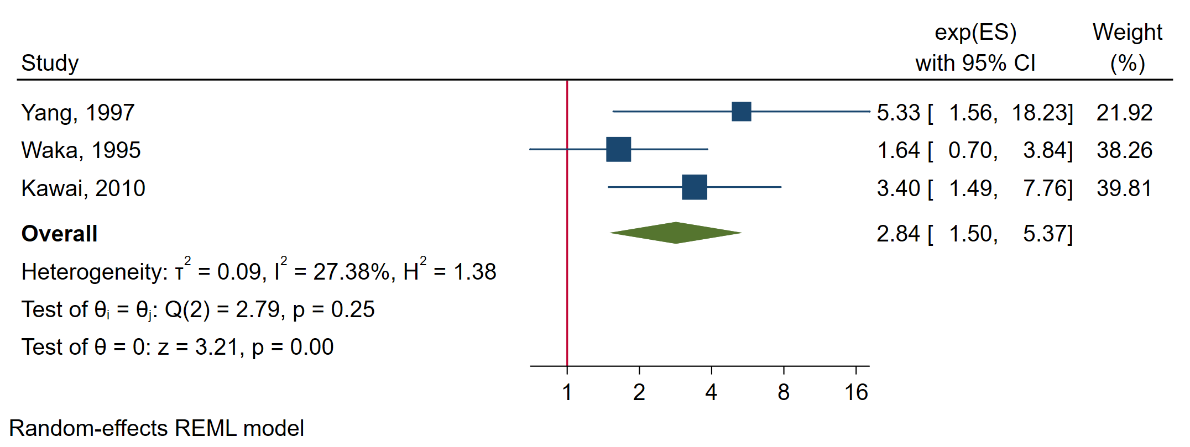


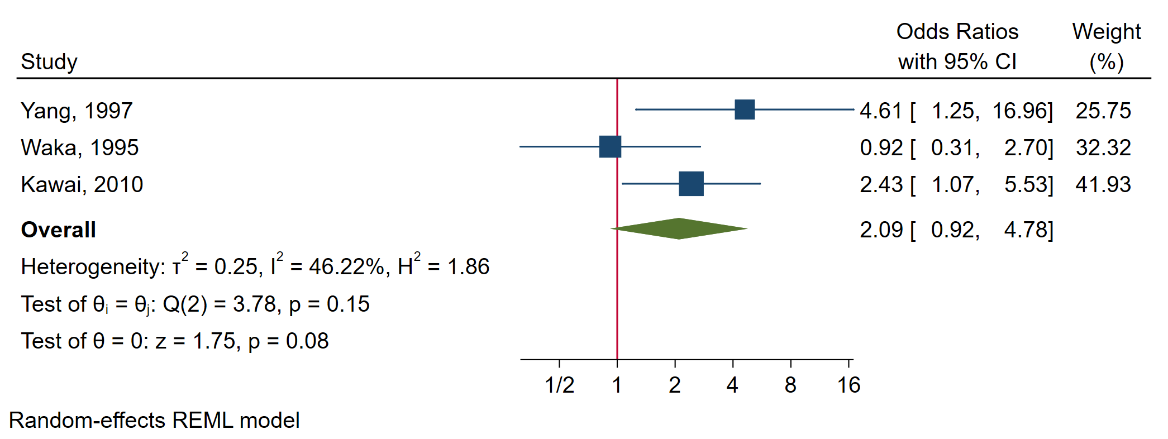


**Figure S6** Breast cancer risk comparison for pre-menopausal women (top figure) and post-menopausal women (bottom figure) with a family history in any relative


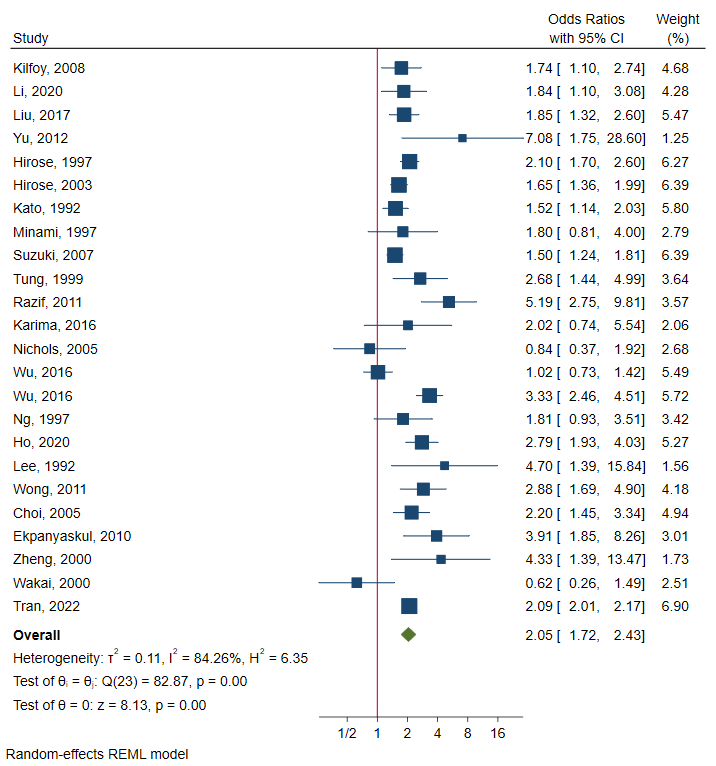


**Figure S7** Breast cancer risk for women with a family history in any relative in East and Southeast Asia


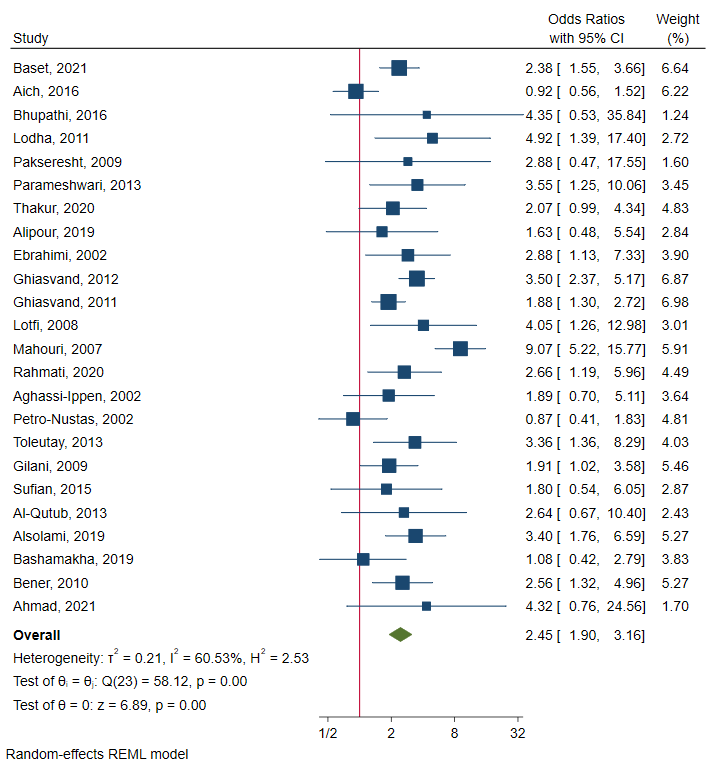


**Figure S8** Breast cancer risk for women with a family history in any relative in the rest of Asia apart from East and Southeast Asia


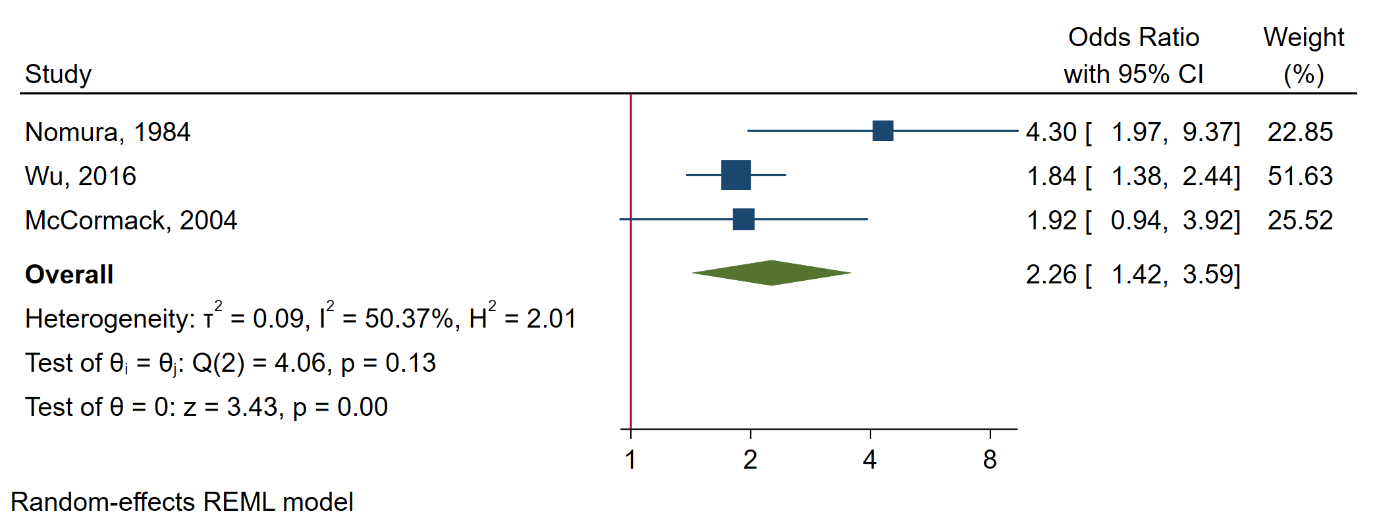


**Figure S9** Breast cancer risk for women living in non-Asian countries with a family history in any relative


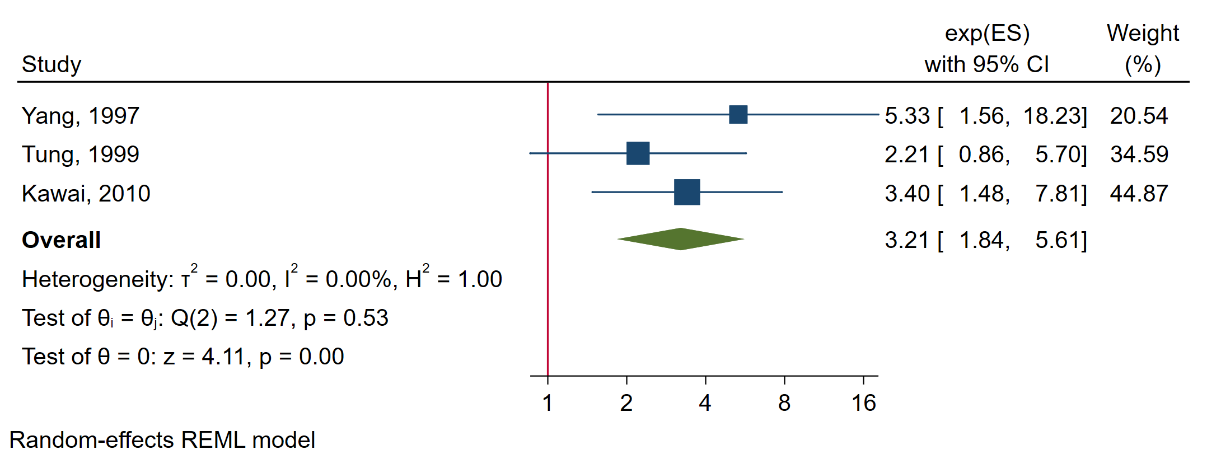


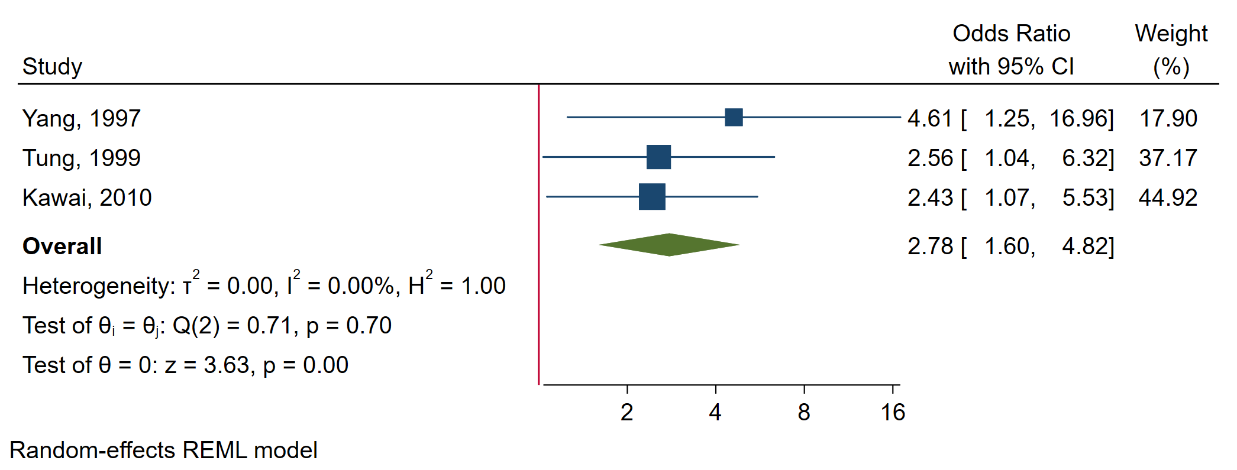


**Figure S10** Breast cancer risk comparison for pre-menopausal women (top figure) and post-menopausal women (bottom figure) with a family history in a first-degree relative


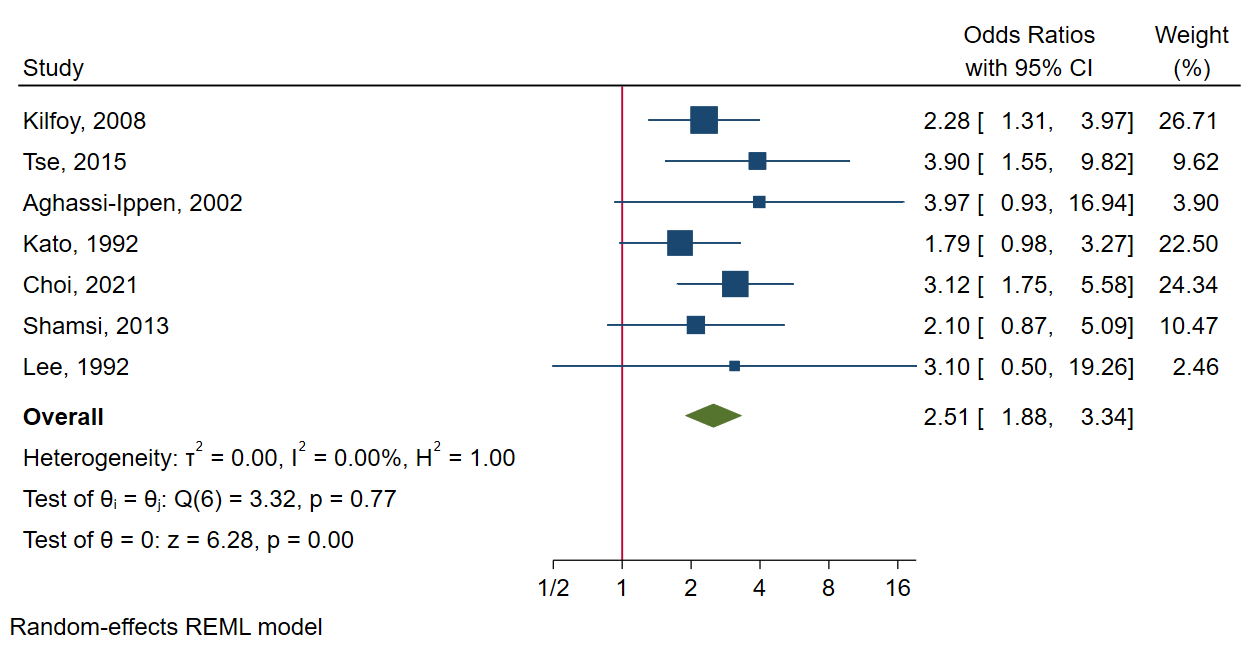

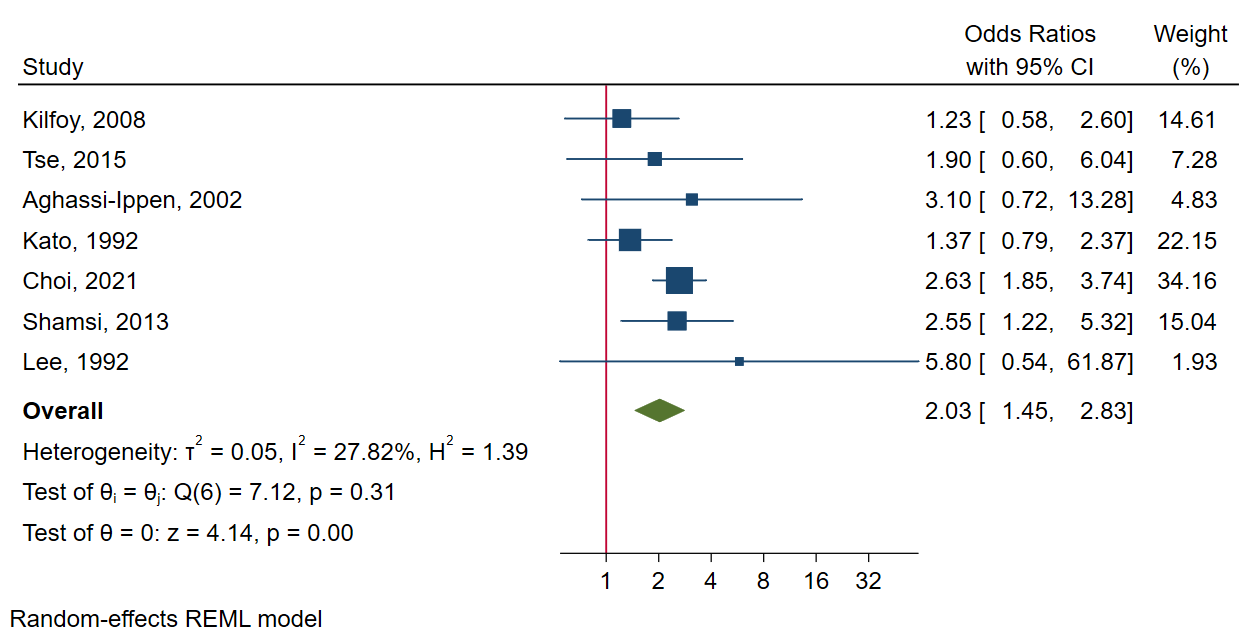


**Figure S11** Breast cancer risk comparison for women with a family history in mothers (top figure) and a family history in sisters (bottom figure)


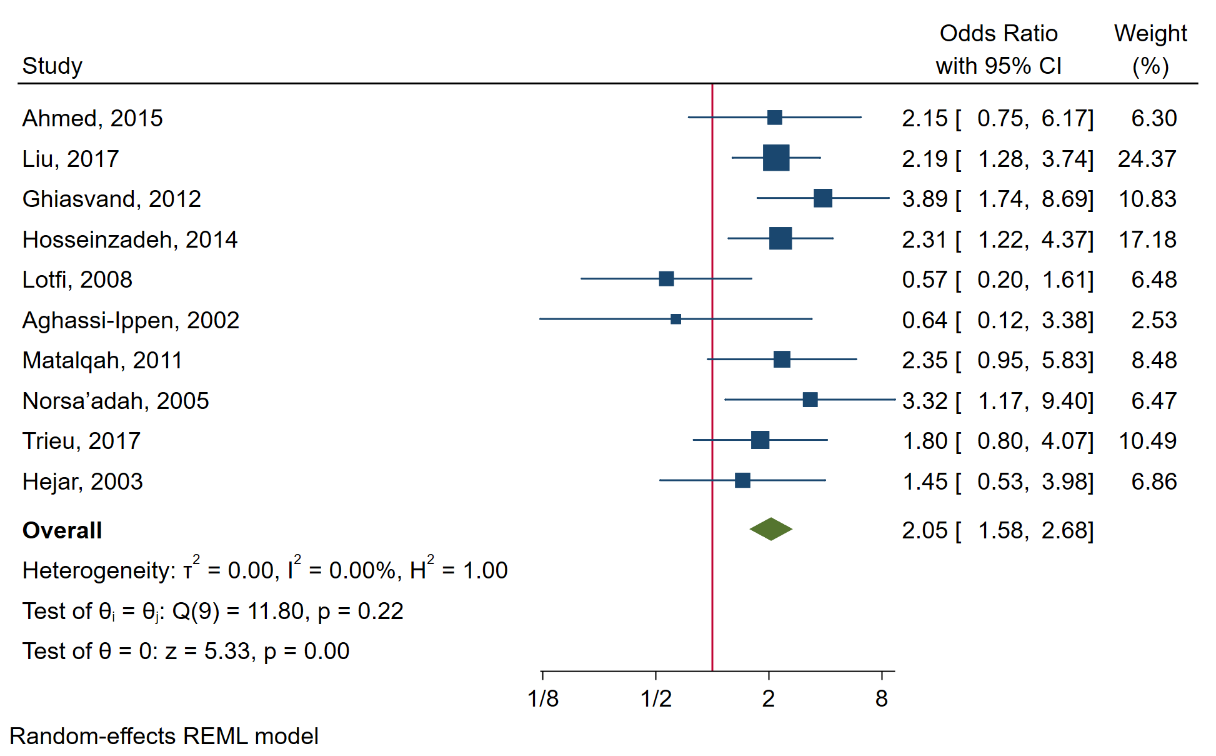


**Figure S12** Breast cancer risk for women with a family history in a second-degree relative


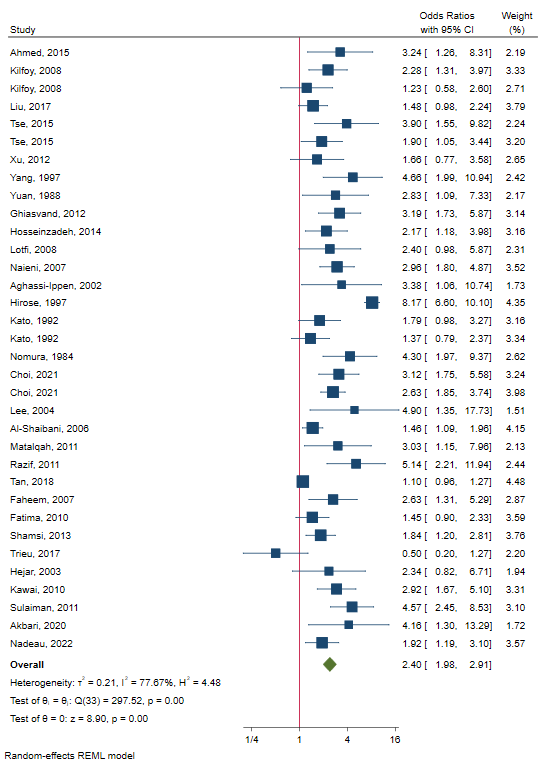


**Figure S13** Breast cancer risk for women with a family history in a first-degree relative from the sensitivity analysis


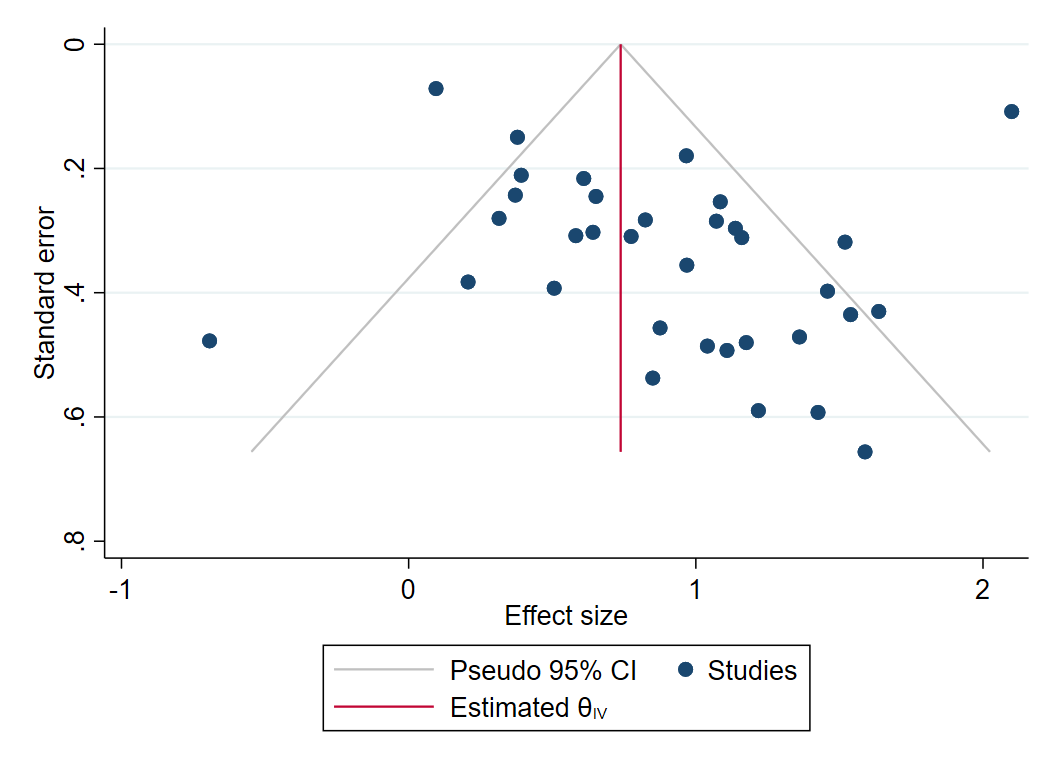


**Figure S14** Funnel plot for the breast cancer risk associated with a family history in first-degree relatives from the sensitivity analysis
